# Variation of tap-water isotope ratios and municipal water sources across Kyiv city, Ukraine

**DOI:** 10.1007/s43832-022-00021-x

**Published:** 2022-11-07

**Authors:** Elizabeth Avery, Olena Samonina, Iryna Vyshenska, Alan E. Fryar, Andrea M. Erhardt

**Affiliations:** 1 Department of Earth and Environmental Sciences, University of Kentucky, 101 Slone Research Building, 121 Washington Ave, Lexington, KY 40506 USA; 2 Department of Environmental Sciences, National University Kyiv-Mohyla Academy, Kyiv, Ukraine; 3 Institute for Advanced Studies, Technical University of Munich, Munich, Germany

**Keywords:** Isotopes, Hydrogen-2, Oxygen-18, Tap water, Climate change, Ukraine

## Abstract

Stable isotopes of water allow researchers to examine water pathways and better understand spatial and temporal variability in mixtures of municipal water sources. In regions such as Kyiv (Ukraine), with a water supply that is vulnerable to the effects of climate change, pollution, and geopolitical conflict, such understanding is critical for effective water management. Trends in stable isotope values and water sources can function as a confirmation of municipal data. Additionally, these data can provide an early signal for the effects of climate change on these sources, reducing uncertainty from physical measurements.

For this study, tap water, surface water, and groundwater were collected over 14 months in Kyiv and nearby Boryspil, Brovary, and Boyarka and measured for hydrogen (δ^2^H) and oxygen (δ^18^O) stable isotopes. The stable isotope values from the tap water for each district show a general seasonal trend in water sources, with more groundwater used in the supply in the winter for most districts. Spatially, groundwater use increases from south to north in the left-bank districts in Kyiv city and groundwater use generally decreases from south to north in the right-bank districts. As precipitation patterns shift and temperatures increase, the reliance on particular water sources may need to shift as well. Overall, δ^2^H and δ^18^O data provide a baseline expectancy for current water use throughout the year and, from this, deviations can be assessed early.

**Supplementary Information:**

The online version contains supplementary material available at 10.1007/s43832-022-00021-x.

## Introduction

### Study rationale

Stable isotopes of water have been used to characterize evaporation of surface water [1–3], groundwater recharge [4–6], seasonal changes to streamflow [2, 3, 5, 7], and precipitation patterns [5, 8]. Stable isotope ratios have also been used during tap-water surveys to characterize input from complex water sources [9, 10] and spatial/temporal patterns in supply [9, 11–13]. Numerous studies have incorporated stable isotopes of tap water to investigate patterns of water sources and timing of these sources, from local and regional to country-wide scales e.g. [9, 11, 12, 14–20].

Tap-water surveys can identify changes to water sources with climate change and confirm the mixture of these sources in the water supply. While many cities have well-documented sources and timings of changes between sources, relying only on geospatial analysis and field measurements can introduce uncertainty. Additionally, there can be significant water loss between the intake of water and delivery to individual homes. Stable isotope analysis can delineate and quantify sources and mixtures based on the collection of input samples (surface water and/or groundwater) and output samples (tap water). Analysis of these samples can yield monthly, seasonal, or spatial patterns and can help confirm or add information about water supply sources [9].

Characterization of spatial and temporal patterns of water supply is critical, especially in regions vulnerable to climate change [9]. In Ukraine, the risk of widespread drought has increased [21, 22] and it is predicted, using Representative Concentration Pathway (RCP) scenarios and the global climate model GFDL-ESM2M, that winter temperatures in northern Ukraine could rise by 3.2 °C by 2070 and by 5.0 °C by 2100 [23, 24]. As climate change progresses there will likely be an impact on water resources in Kyiv, as 89% of the city’s water supply is from surface-water sources (the Dnipro and Desna rivers) and 11% is from groundwater on average [25]. Furthermore, changes in timing and amount of precipitation can lead to flooding or drought, affecting the quantity and quality of water [26, 27]. For a city that heavily relies on surface water, it is critical to understand spatial and temporal patterns in the municipal water supply.

For this study, tap water, groundwater, and surface water samples were collected over a period of fourteen months in Kyiv (November 2019 through December 2020). While studies exist that examine tap water against both groundwater and surface water, these are more often regional or country-scale e.g. [17, 28, 29] rather than metropolitan studies e.g. [9, 11, 30, 31] which examine sources to sections of the city and the surrounding regions in detail. Kyiv receives the majority of its water from surface water sources and is also vulnerable to the effects of climate change [32]. Therefore, it is critical to understand current trends in the water supply and how these may change in the future, specifically as increasing temperatures potentially shifts the timing and amount of precipitation.

### Study location

**Fig. 1:**
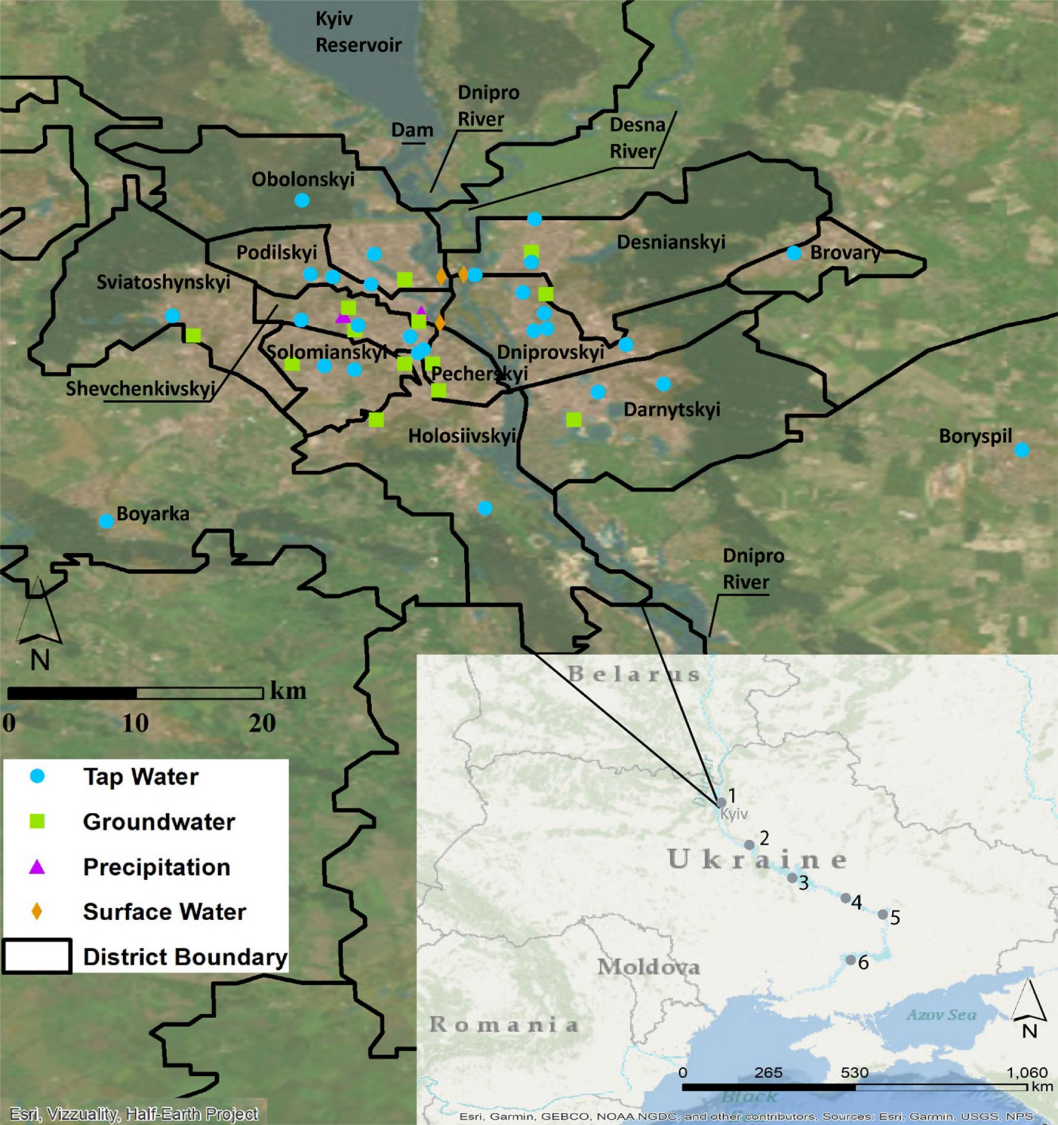
Study location. Kaniv Reservoir and its dam (not pictured) are located approximately 50 km and 85 km south of Kyiv city, respectively (or within and 9 km south of Kyiv oblast, respectively). Tap water sampling locations are shown by blue circles, groundwater sampling locations by green squares, precipitation collection locations by purple triangles, and river sampling locations by orange diamonds. On the inset map, reservoir locations along the Dnipro River are indicated by a gray circle and a number (1 = Kyiv,  2 = Kaniv, 3 = Kremenchutske, 4 = Kamianske, 5 = Dniprovske, and 6 = Kakhovske). Base maps from ESRI and district shapefile from R. Hijmans [33–35]

Kyiv, with a population of 2.9 million, is divided into ten administrative districts (Desnianskyi, Dniprovskyi, Darnytskyi, Obolonskyi, Podilskyi, Sviatoshynskyi, Shevchenkivskyi, Solomianskyi, Pecherskyi, and Holosiivskyi districts; Fig. 1). The population of each district varies from 163,672 in Pecherskyi to 384,616 in Solomianskyi. The districts with a population above 300,000 are Desnianskyi, Dniprovskyi, Darnytskyi, Obolonskyi, Sviatoshynskyi, and Solomianskyi. Districts with a population between 200,000 and 300,000 are Podilskyi, Shevchenkivskyi, and Holosiivskyi; Pecherskyi is between 100,000 and 200,000 [36].

Kyiv is located in the Forest-Steppe region [37] and is within zone Dfb of the Köppen-Geiger climate classification, which is humid with snowy winters and warm summers [38]. Historically, the average temperature for the warm season is 19 °C and the cold season is − 3 °C [39], though in the year 2020 these temperatures increased [40]. Historically, the maximum precipitation occurs in July and the minimum in February, with an average annual precipitation total of 651 mm [39]. However, this also shifted in 2020, with the maximum occurring in May and the minimum occurring in November, with an annual precipitation total of 564 mm [40].

The Dnipro River, one of the two rivers that supply surface water to the city water supply, separates Kyiv into the right and left banks, with three districts (Desnianskyi, Dniprovskyi, and Darnytskyi) on the left bank and seven (Obolonskyi, Podilskyi, Sviatoshynskyi, Shevchenkivskyi, Solomianskyi, Pecherskyi, and Holosiivskyi) on the right. Dams along the Dnipro River, notably the Kyiv and Kaniv dams in the vicinity of the study area, create reservoirs [41]. The average temperature of the Dnipro River has increased, and winter ice cover and thickness have decreased, after the construction of the Kyiv and Kaniv Reservoirs (north and south of Kyiv city, respectively) [42, 43]. For water supply from the Kyiv Reservoir there are two withdrawals per day during winter, one in the morning and one in the evening, and only one in the evening during the summer [42]. The Desna River, the other surface water source for tap water in Kyiv, meets the Dnipro River at the north of the city (Fig. 1).

The ten districts in Kyiv use differing water source percentages between surface water and groundwater to supply drinking water to the population via tap water. In the city, the water supply is 339.6 million m^3^/hour. This includes both the sale of water (283.1 million m^3^/hour, which includes 236.4 million m^3^/hour for the total population of the city) and lost/unaccounted-for water (56.5 million m^3^/hour) [25]. As of 2013, the available groundwater for drinking and industrial water in the Upper Dnipro River Basin was 1,137,990 m^3^/day [41].

## Methods

### Sample collection

From November 2019 through December 2020, tap-water samples were collected from each district in Kyiv, Ukraine and three cities in the Kyiv oblast (province) (Boryspil, Boyarka, and Brovary). Cold water samples were collected after allowing the faucet to run for at least 30 s to clear any standing water from the pipes. Volunteers were recruited through students from National University Kyiv-Mohyla Academy and were provided training in-person and via video; all sampling supplies were provided. Samples were collected during the final week of each month, with the majority on the final day or two of the month, and from the same locations each month when possible (some disruptions occurred due to COVID-19 restrictions). Any changes in locations were noted and considered when calculating the isotopic average and percentage contributions from sources.

Groundwater samples were collected most months from public water wells located in each district. Only wells with stable groundwater isotopic signals were used since an unstable signal indicates potential leaks in the pipes. Stability was defined as remaining within ranges of 4.5‰ and 0.65‰ for δ^2^H and δ^18^O, respectively, though most locations were well below these thresholds. More than one well from each district was sampled throughout the year when possible and locations where the isotopic signal was not stable were discarded. If both locations were isotopically stable, then both locations were included in the dataset. Wells were in constant use immediately before collection so standing water in the pipes should not have been an issue.

Surface-water samples were collected each month from the Dnipro and Desenka Rivers. During the months of March, May, and June the sample location was changed due to COVID-19 restrictions and disruptions to public transit. Most months samples were collected with weighted bottles from the middle of the river at Pivnichnyi Bridge. In March, May, and June, samples were collected by wading approximately 3 m from the banks at Park Bridge (Dnipro River only). Sample locations are shown on Fig. 1.

All samples were collected in 40- to 60-mL HDPE bottles, sealed with Parafilm, and stored in a refrigerator until analysis except during transport from Ukraine to the United States.

### Stable isotope analysis

Samples were analyzed at the University of Kentucky using a Los Gatos T-LWIA-45-EP liquid water isotope analyzer. All water samples were first filtered with sterile 0.45-μm filters. Samples were then injected via autosampler nine times, with the first four injections ignored to mitigate between-sample memory effects. The raw hydrogen and oxygen isotopic data were then normalized to the VSMOW-SLAP (Vienna Standard Mean Ocean Water-Standard Light Antarctic Precipitation) scale using two different certified standards with contrasting isotopic values: USGS49 Antarctic Ice Core Water (δ^2^H_VSMOW-SLAP_ = –394.70‰, δ^18^O_VSMOW-SLAP_ = –50.55‰) [44] and USGS50 Lake Kyoga Water (δ^2^H_VSMOW-SLAP_ =  + 32.80‰, δ^18^O_VSMOW-SLAP_ =  + 4.95‰) [45]. Multiple in-session measurements of a third standard, USGS45 Biscayne Aquifer Drinking Water (δ^2^H_VSMOW-SLAP_ = –10.30‰, δ^18^O_VSMOW-SLAP_ = –2.24‰) [46], were used to evaluate the precision and accuracy of the isotopic data, with a long-term standard deviation of 0.16 ‰ for δ^2^H and 0.08 ‰ for δ^18^O over 120 analyses.

### Water source percentage calculation

Using IsoSource [47], all possible percentages of contributions to tap water from groundwater, the Dnipro River, and the Desenka River (which receives water from the Desna River) were calculated based on the δ^2^H and δ^18^O values from the input (groundwater and the river samples) and the output (tap water samples from each district). This was repeated for each district each month of the year 2020. IsoSource calculates the source contributions through a linear mixing model. The contributions are calculated in increments designated by the user (in this study 1%) and the calculated contributions sum to 100%. IsoSource will report all calculated contribution percentages that fall within the mass balance tolerance, so multiple possible solutions may be reported. Since all solution combinations are equally likely, it is common practice to report all the possible solutions as a range [48], as in this study.

Data were not available detailing the depth of groundwater collection or supply in any district. To account for this unknown, both high and low groundwater isotope values were used each month for the entire right bank and the entire left bank. This means that the highest and lowest groundwater isotope value for the left bank was used for contribution calculations for Desnianskyi, Dniprovskyi, and Darnytskyi districts and the highest and lowest groundwater isotope value for the right bank was used for contribution calculations for Obolonskyi, Podilskyi, Sviatoshynskyi, Shevchenkivskyi, Solomianskyi, Pecherskyi, and Holosiivskyi districts.

IsoSource was also used to estimate the percentages of contributions to the Dnipro River from groundwater and precipitation for each month of the year 2020. Stable isotope values of groundwater from the Podilskyi and Dniprovskyi districts were used as the groundwater inputs since these were the districts nearest to the river sampling location.

### Mean transit time estimation

Because groundwater contributes to the flow of the Dnipro River, it is necessary to consider the importance of this contribution with the possibility of future stress on water resources due to climate change. To use the isotopic data in IsoSource, it was first necessary to calculate mean transit time to determine the time period for which precipitation data should be selected. For instance, if the mean transit time is 6 months, then precipitation data from 6 months prior to the collection of the groundwater and Dnipro River data was used as an input.

Due to the last hydrograph data available for this region being from 2015 [49], mean transit time has been estimated only. Mean transit time for the Dnipro River was estimated using the stable isotope values from precipitation and river water, along with a hydrograph from 2015 downstream from Kaniv Reservoir south of Kyiv [49], using methods from Dosa et al. [50]. To calculate mean transit time from the stable isotopes, the equation used is

$${\tau }_{r}= \frac{\sqrt{\frac{1}{{f}^{2}}-1}}{2\uppi }$$

where τ_r_ is mean transit time in years and *f* is the amplitude damping. Amplitude damping, f, is found from the amplitude of δ^18^O in runoff divided by the amplitude of δ^18^O in precipitation. To calculate mean transit time from the hydrograph, the equation used is

$${\tau }_{c}= \frac{2{t}_{r}}{L}{e}^{\lambda }$$

where τ_c_ is mean transit time, t_r_ is the total hydrograph recession time, L is the maximum flow path length, and e^λ^ is the topographic index. The total hydrograph recession time, t_r_, and the maximum flow path length, *L*, were estimated from Fig. 2 of Obodovskyi et al. [49]. The topographic index, e^λ^, was estimated from the slope at Hidropark (0 to 8%) given in Pozharska [51] and a catchment area of 90,090 km^2^ at Kyiv Reservoir, taken from the River Basin Management Plan [41].

**Fig. 2:**
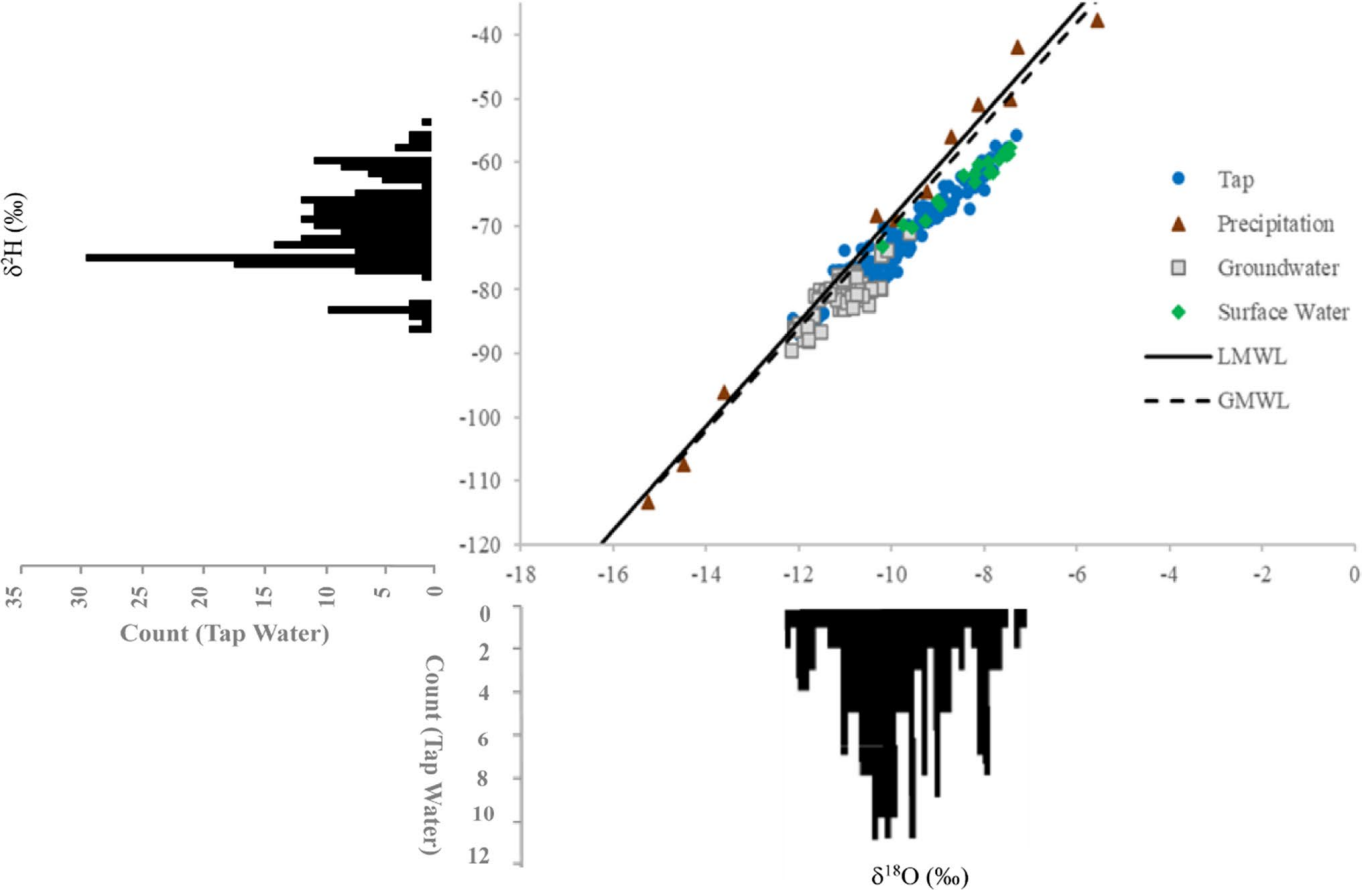
Stable isotope (δ^2^H and δ^18^O) values of tap water. In the upper right, δ^2^H and δ^18^O values of tap water, precipitation, groundwater, and surface water are plotted against the global meteoric water line (GMWL) [8] and local meteoric water line (LMWL) [40] for Kyiv. At the upper left and lower right, the δ^2^H and δ^18^O values, respectively, are shown in histograms for the tap water samples

## Results

### Stable isotopes

**Fig. 3:**
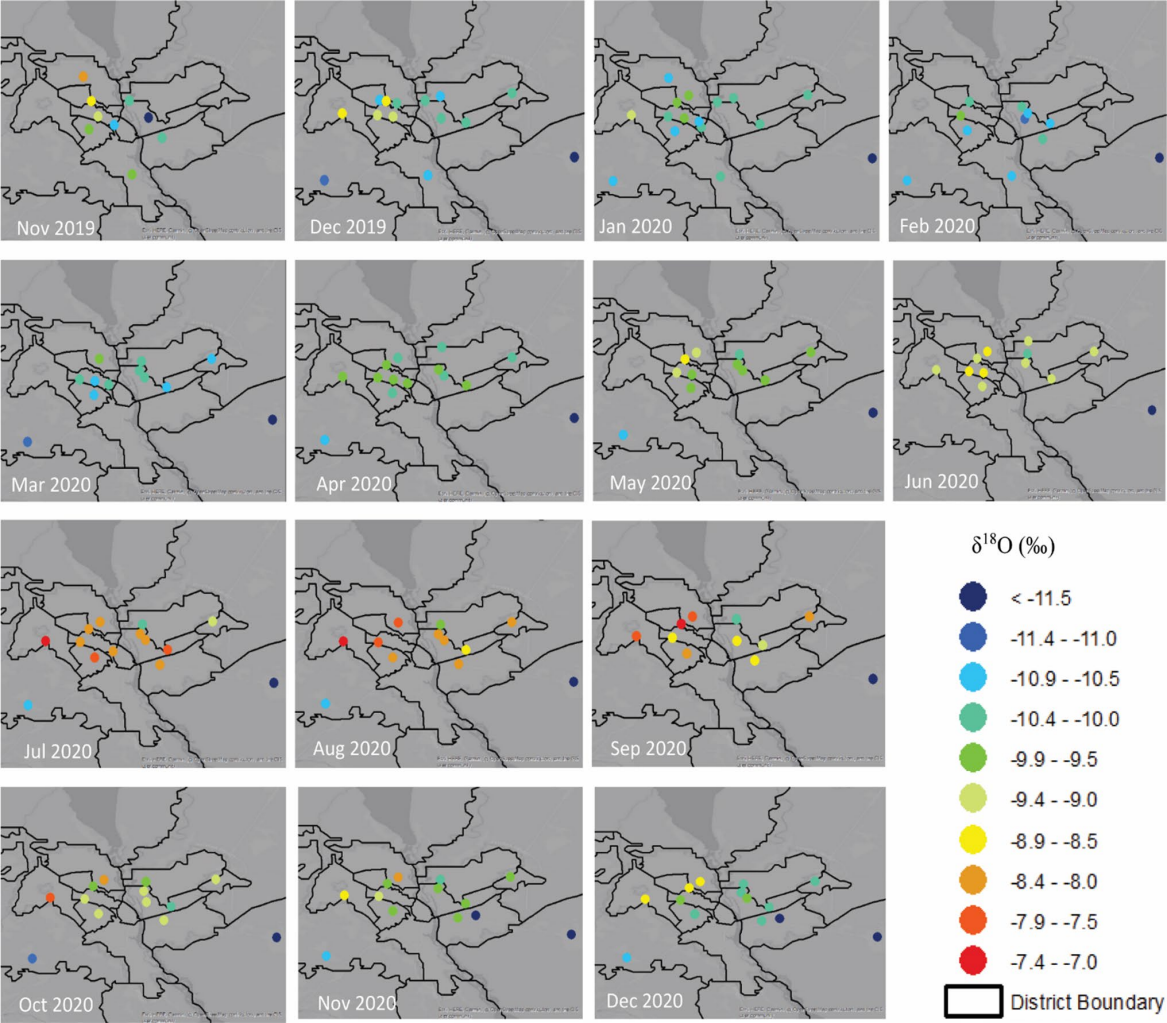
Spatial and temporal patterns of δ^18^O of tap water samples [34, 52]

For the ten districts and three cities where tap-water samples were collected, stable isotope values ranged from − 87.9‰ to − 55.8‰ for δ^2^H and from − 12.1‰ to − 7.3‰ for δ^18^O (Figs. 2, 3; Additional file 1: Table S1). Generally, for all districts and the city of Brovary, stable isotope values were the most negative in the winter and spring and the least negative in the summer and fall. The exception to this trend is the isotope values of the tap water from the Desnianskyi district, which were most negative in winter and fall and the least negative in spring and summer. For the districts of Darnytskyi, Pecherskyi, and Holosiivskyi, lower coverage meant that some seasons did not have enough samples to calculate averages. However, both Darnytskyi and Pecherskyi had enough samples to calculate three of the seasons, with winter the most negative and summer the least negative. The cities of Boryspil and Boyarka were seasonally invariant due to tap water being supplied by groundwater at the location sampled.

Stable isotope values from surface water collected in Kyiv ranged from − 73.2‰ to − 57.6‰ and − 63.2‰ to − 58.2‰ for δ^2^H and from − 10.2‰ to − 7.4‰ and − 8.2‰ to − 7.5‰ for δ^18^O from the Dnipro River and Desenka River, respectively (Additional file 1: Table S2). Surface water samples collected from the Dnipro River in Cherkasy (~ 190 km south of Kyiv) had similar stable isotope values, from − 70.5‰ to − 56.3‰ for δ^2^H and from − 9.8‰ to − 7.2‰ for δ^18^O. Groundwater collected from each district showed relatively consistent isotope values throughout the year. These values ranged from − 89.5‰ to − 71.2‰ for δ^2^H and from − 12.2‰ to − 9.6‰ for δ^18^O (Additional file 1: Table S3). While stable isotope values from the groundwater plot near the global meteoric water line (GMWL), similar to the precipitation samples (Fig. 2), those from the tap water and surface water samples plot below the GMWL, indicating alteration of the original isotopic signal by evaporation [53].

### Source contributions to tap water

Calculated source contributions to tap water, determined using the IsoSource mixing model, are compiled in Table 1. The contribution of groundwater to tap water from each district generally was the highest in the fall and winter months, with the highest contribution from surface water in late spring and summer. For this study, a majority contribution is considered at least 67%. Since there can be large ranges of possible contribution percentages, a 2/3 majority was chosen to indicate a clear groundwater or surface water signal rather than simply anything over half. A majority contribution from surface water is more typical than a majority contribution from groundwater for nearly all districts, which is expected due to a higher reliance on surface water as a drinking water resource in this region. Incidences of majority contributions from groundwater or surface water are identified with bold numbers in Table 1. Two notable exceptions were tap water from Sviatoshynskyi with a majority contribution from surface water for the entire year and Desnianskyi with no clear majority contribution throughout the year (Table 1). Pecherskyi and Holosiivskyi districts did not have enough samples collected to make detailed observations for the entire year.

**Table 1:** Percentage groundwater and surface water contribution to district tap water by month.

District	Month	Groundwater (%)	Surface water (%)
Desnianskyi	Dec-19	***74***	26
	Jan-20	60–66	34–40
	Mar-20	***100***	0
	Apr-20	46–51	49–54
	May-20	16–20	***80–84***
	Jun-20	14–20	***80–86***
	Jul-20	59–62	38–41
	Aug-20	51	49
	Sep-20	62–66	34–38
	Oct-20	62–66	34–38
	Nov-20	***63–71***	29–37
	Dec-20	***95–100***	0–5
Dniprovskyi	Dec-19	42–43	57–58
	Jan-20	35	65
	Feb-20	34	66
	Mar-20	20–23	***77–80***
	Apr-20	36–41	59–64
	May-20	25–28	***72–75***
	Jun-20	3–6	***94–97***
	Jul-20	8	***92***
	Aug-20	17–24	***76–83***
	Sep-20	50–52	48–50
	Oct-20	36–57	43–64
	Nov-20	50	50
	Dec-20	41	59
Darnytskyi	Feb-20	50–51	49–50
	Jul-20	0	***100***
	Aug-20	12–17	***83–88***
	Sep-20	46–49	51–54
	Oct-20	35–39	61–65
	Nov-20	36	64
	Dec-20	41	59
Obolonskyi	Jan-20	***67–72***	28–33
	Mar-20	37–43	57–63
	Apr-20	0	***100***
	May-20	0	***100***
	Jun-20	2–5	***95–98***
	Jul-20	0	***100***
	Aug-20	0	***100***
	Sep-20	9–12	***88–91***
	Oct-20	5–9	***91–95***
	Nov-20	0	***100***
	Dec-20	0–3	***97–100***
Podilskyi	Dec-19	56	44
	Jan-20	1	***99***
	Feb-20	***72***	28
	Apr-20	5–10	***90–95***
	May-20	0	***100***
	Jun-20	6	***94***
	Jul-20	0	***100***
	Sep-20	0	***100***
	Oct-20	76	24
	Nov-20	51–52	48–49
	Dec-20	0	***100***
Sviatoshynskyi	Dec-19	0–2	***98–100***
	Jan-20	0	***100***
	Apr-20	0	***100***
	Jun-20	5	***95***
	Jul-20	0	***100***
	Aug-20	0	***100***
	Sep-20	4	***96***
	Oct-20	0	***100***
	Nov-20	4	***96***
	Dec-20	0–3	***97–100***
Shevchenkivskyi	Dec-19	33	***67***
	Jan-20	40–47	53–60
	Feb-20	7–15	***85–93***
	Mar-20	11	***89***
	Apr-20	0	***100***
	May-20	0–6	***94–100***
	Jun-20	4	***96***
	Jul-20	0	***100***
	Aug-20	8–9	***91–92***
	Sep-20	39	61
	Oct-20	34–52	48–66
	Nov-20	37	63
	Dec-20	***86–87***	13–14
Solomianskyi	Jan-20	48–59	41–52
	Feb-20	42	58
	Mar-20	38–41	59–62
	Apr-20	***68***	32
	May-20	65–66	34–35
	Jun-20	8–15	***85–92***
	Jul-20	10–13	***87–90***
	Aug-20	12–14	***86–88***
	Sep-20	30–33	***67–70***
	Oct-20	39–44	56–61
	Nov-20	44–53	47–56
	Dec-20	***76–99***	1–24

Majority contribution (above 67%) is noted in bold italics. At times there may be multiple unique solutions in IsoSource and these are reported as a range of values (e.g. 60–66% groundwater contribution for Desnianskyi district in January 2020)

### Dnipro River mean transit time

River transit times are necessary to quantify the groundwater and precipitation contributions to surface waters and to attribute the correct isotope value to source calculations. The mean transit time estimated at Kaniv through isotope versus hydrograph methods varies, with the stable isotope values resulting in approximately 6-month transit times, while hydrographs estimated 9 months. The discrepancy between the two methods is normal, as the hydrograph method depends on the interpreted length of the falling limb of the hydrograph [50]. Because the estimate in this study used a different year and location (south of Kyiv rather than north of Kyiv) for the hydrograph method based on available data at the time of publication, this discrepancy is expected. For instance, in Dosa et al. [50], the isotope method and the hydrograph recession method could have a difference of up to 30 months using the longer hydrograph recession interpretation or up to 10 months using the shorter hydrograph recession interpretation. In this study, the shorter hydrograph recession interpretation was used to estimate the 9-month mean transit time, while a longer hydrograph recession interpretation yielded a mean transit time of 13 months for the Dnipro River.

Additionally, as only one year of stable isotope values for precipitation exists for Kyiv, this presented more uncertainty for estimating precipitation contribution to the river in the first half of 2020 (since data from 2019 do not exist). For the purpose of this estimation, it was assumed the stable isotope values of precipitation for 2019 would be similar to the results collected in 2020. While significant interannual variation in values is possible [53], our estimation yielded similar results to the groundwater contribution percentage stated in the River Basin Management Plan [41]. This supports the use of 2020 data as a proxy for 2019, at least within the objectives and resolution of this study.

## Discussion

### Temporal and spatial patterns

In general, districts on the left bank used a higher percentage of groundwater during the year, though temporal patterns were similar to districts on the right bank (for instance, higher percentage of groundwater use in the winter and a lower percentage of groundwater use in the summer). This pattern also varied spatially, with Desnianskyi district generally using the highest percentage of groundwater and Darnytskyi using the lowest percentage of groundwater on the left bank. Desnianskyi district’s large contribution from groundwater is also indicated in the temporally different pattern in the stable isotopes from tap water, which are most negative in winter and fall. This agrees with the timing of the greatest contribution of groundwater to the tap water, since, across Kyiv, the isotope values in the groundwater are more negative than those in the surface water during the entire year. The other districts more closely follow the precipitation trend of most negative stable isotope values in winter and spring and least negative in summer and fall. The tap water from these districts also receives more surface-water contributions throughout the year as compared to Desnianskyi district.

**Fig. 4:**
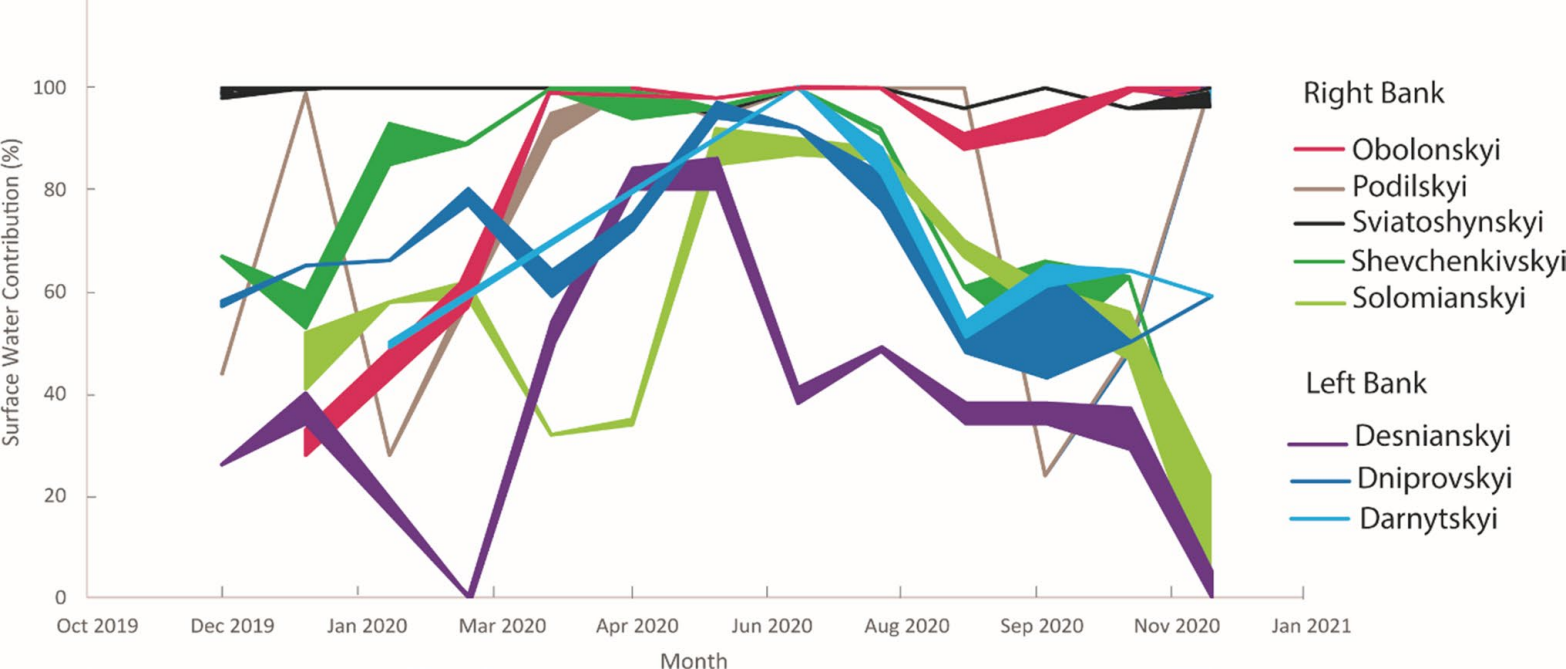
Percent range of surface water contribution to tap water each month by district. The width of the line represents the range of possible contribution percentages given by IsoSource (for instance, a wider line represents a larger range of possible contribution percentages, while a thin line represents fewer possibilities)

There is both more temporal and spatial variability on the right bank, including seasonal variability in surface versus groundwater sources. With the exception of Pecherskyi, all districts on the right bank use a lower percentage of groundwater than those on the left bank. Obolonskyi, Podilskyi, and Sviatoshynskyi have the greatest surface water contribution, with < 10% groundwater contribution percentage at least half the year (Fig. 4, Table 1). However, the groundwater contribution in these districts increases up to 76% briefly during the fall or winter. Tap water from Shevchenkivskyi district also shows a strong influence from surface water (Fig. 4, Table 1), but with a more consistent groundwater signal of more than 10% for most of the months of the year. Neighboring Solomianskyi shows the expected pattern of higher percentage of groundwater contribution in the winter with a gradual decrease in percentage to the summer and then a gradual increase again leading into fall and winter. The percentage pattern is similar to that of the districts on the left bank (Fig. 4, Table 1). Spatial patterns on the left bank show a decrease in reliance on groundwater from north to south, while the opposite is generally true for the right bank. However, due to COVID-19-induced difficulties in travel, samples for Pecherskyi and Holosiivskyi districts were only collected in the first half of the year, so the most southern portions of the right bank are not included in this assessment.

Outside of Kyiv city and the study boundary, but within Kyiv oblast, tap water from Boryspil and Boyarka were seasonally invariant, consistent with a groundwater source. These samples came from sampling locations with a groundwater well, not municipal tap water, and were excluded from analysis trends. However, isotope values of tap water from Brovary showed a similar pattern to those from Desnianskyi district. While groundwater samples were not collected from Brovary, it is expected that, based on the stable isotope values and the city’s proximity to the district, groundwater and surface water percentages would be similar to those of Desnianskyi district.

The stable isotopes of the groundwater samples also showed some slight spatial patterns, with more enriched ^2^H and ^18^O values in groundwater samples collected from districts generally in the northern portion of the city and mostly along the Dnipro River. It is possible that the proximity to the river and the Kyiv reservoir could influence these values if river water is infiltrating into the aquifer. However, groundwater from Boyarka had similar stable isotope values to those from Obolonskyi, Pecherskyi, and Dniprovskyi, despite its location approximately 20 km from the Dnipro River. Locations with groundwater samples that were more depleted in ^2^H and ^18^O were generally in the southern portion of the city and/or farther from the Dnipro River. These patterns occurred both on the right and left bank, though the elevation ranges from 89 to 208 m above sea level (asl) [40], with the right bank being higher elevation than the left. While it is possible that the Dnipro River influences groundwater in the districts nearby, based on the similarity of groundwater from Boyarka, it is likely that the main driver of differences in stable isotope values among the groundwater samples is retrieval from different depths. However, as it was not possible to determine the depths of the public wells, this cannot be certain and should be determined in future studies in this area.

**Fig. 5:**
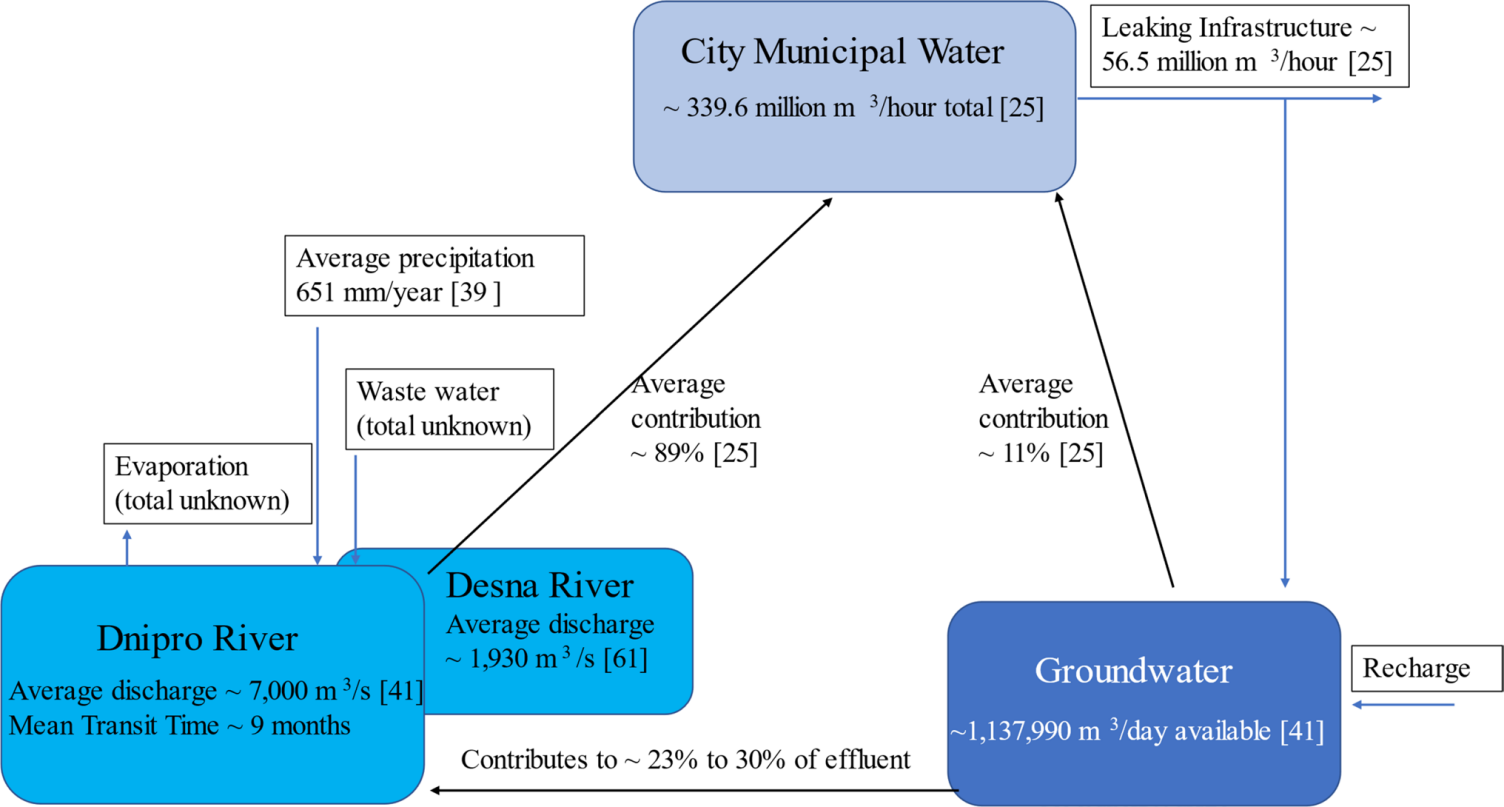
Schematic of sources to Kyiv water supply. Shown are inputs to the sources and amounts if they are publicly known, percent contribution of the sources to the water supply, and amount output from the supply, including losses from leaking infrastructure [25, 39, 41, 61]

The data collected improve our understanding of water use at a seasonal and/or oblast scale, particularly since this information was not publicly available at the time of this study, while highlighting regions more susceptible to changes in water supply. The Dnipro and Desna Rivers rely on runoff from snow and precipitation each year, as well as contribution from groundwater (Fig. 5). As climate change progresses and the timing and amount of precipitation shift [40], water managers may have to adjust the timing and amount of surface water used in each district. Since shifts in precipitation patterns will also affect the timing and amount of recharge to groundwater [29], stable isotope analysis of inputs and outputs to the municipal water supply can provide critical early warning of changes that physical measurements could take longer to identify due to uncertainties [9].

In Kyiv, the effects of climate change have already become apparent. Boychenko et al. [54] analyzed long-term meteorological data for the past 100 to 130 years from 25 stations across Ukraine. Those authors found that the annual temperature had increased by 1.0 ± 0.2 ℃ per 100 years in the northern and northeast regions, and that there has been an increase in cold-season temperature of 1.0–2.0 ℃ per 100 years and a decrease of 10–15% in annual precipitation for the northern and northwest regions. Besides rising air temperatures and changes in precipitation patterns, the average temperature of the Dnipro River has also increased and winter ice has decreased [42]. The thinning and disappearance of winter ice coincides with the timing of the highest groundwater withdrawals for the majority of the districts. As the ice continues to decrease, this could make more surface water available in the winter. The potential shift in timing of surface water availability may require less adjustment from districts that commonly use more surface water throughout the year if this increases availability in the winter.

For the Dnipro and Pripyat river basins, under Representative Concentration Pathway (RCP) 2.6, mean annual discharge is predicted to decrease by as much as 20% for both 2041–2070 and 2071–2100, though the Desna river basin shows a lesser change (from − 10% to + 6%) during these same time periods [55]. Under RCP 8.5, mean annual discharge is projected to decrease nearly 25% for the Dnipro River and more than 25% for the Pripyat River, while the Desna River is expected to have either a very small positive change or no change [55]. The decrease predicted for the Dnipro and Pripyat rivers is expected for the entire year, except in some cases during February–March [55], which means that there may be some adjustment in using the Dnipro for a municipal water source. As less surface water is available for use, districts that rely more on this source year-round, such as Sviatoshynskyi, may need to utilize more groundwater. Both these scenarios illustrate the potential danger in favoring a single water source as climate change progresses in the region.

### Similar studies in Ukraine and worldwide

Stable isotopes of tap water have been studied in another region of Ukraine (Kharkiv). The water supply of this city differs from Kyiv in that tap water comes almost exclusively from surface water, while groundwater is a drinking water source in addition to tap water [6]. In one study at this location, δ^2^H, δ^18^O, and chloride were used to investigate leaks from drinking water and sewage infrastructure. The authors found that urban groundwater was more enriched in ^2^H and ^18^O than rural groundwater, and that surface water sources were more enriched in both isotopes than both groundwater sources. Isotopic enrichment of urban groundwater was attributed to infiltration of precipitation and water of other origins, such as leaking infrastructure [56]. Further studies have continued to investigate this anthropogenic recharge to the groundwater, including identifying sewage source types [6].

Like Kharkiv, Kyiv is an urban environment, and it is possible that similar processes are occurring in both locations. In this study it was also observed that ^2^H and ^18^O values of surface water were more enriched than those of the groundwater. In general, the enrichment seen in the ^2^H and ^18^O values is likely mainly controlled by evaporation from both the reservoir and the more warm/dry (compared to historical values) years of 2019–2020 [40]. Additionally, in drier conditions with intensive evaporation, it is possible that precipitation will not be as effective in recharging groundwater [57]. Since the drier, hotter months are when the precipitation shows a more enriched ^2^H and ^18^O signal [40], it could be also that the groundwater is not receiving as much of this signal. Finally, the urban setting does present some other possibilities. According to the River Basin Management Plan [41], wastewater is discharged into the Dnipro River. The mixing of wastewaters and river waters can also lead to some of the observed enrichment of ^2^H and ^18^O in the surface water [5, 58].

In the USA, Tipple et al. [9] were able to identify not only unique sources to a municipal water supply but also changing of sources over time in the San Francisco Bay area (California), which contains numerous municipalities and different water districts. Similarly, in the Salt Lake Valley region (Utah), Jameel et al. [11] compared municipal water sources for different water districts and noticed a trend towards evaporation over the 3-year study period. Both studies showed temporal and spatial patterns of stable isotopes in tap water, depending on the sources to the water supply, and in the case of surface water sources, seasonal patterns. In Kyiv there is also a temporal and spatial trend in the stable isotopes of tap water, which connected with the changing percentages of surface water and groundwater in the districts’ municipal water supplies.

In the Qinghai-Tibet Plateau (China), Du et al. [28] found that the stable isotope values of tap water could indicate particular sources in the case of a mixed water supply. The stable isotope values of tap water showed spatial patterns across the region and indicated a dominant surface water signal. In the region supplied primarily by groundwater, stable isotope values of tap water did not show seasonal variation. In contrast, in the neighboring region supplied primarily by surface water, seasonal variation was observed in the stable isotope values of the tap water.

In South Africa. West et al. [17] found that the stable isotopes of tap water and groundwater provided spatial patterns that could be predicted by a geostatistical model. The stable isotope values for tap water were similar to those of groundwater or showed an influence of recent precipitation or evaporation of the water source during storage/transport. This study provided a baseline for a region that has not had tap water surveys performed before, as is the case in the current study.

### Groundwater contribution to the Dnipro River

A mean transit time of 6 to 9 months is used to interpret percentage of precipitation and groundwater contributions to the Dnipro River in Kyiv. For 6-month and 9-month mean transit times, the yearly contribution is estimated at 30% and 23%, respectively. The River Basin Management Plan [41] reports that groundwater contribution to the upper Dnipro River is 27%, consistent with the estimated range from this study. Estimated groundwater contributions to the Dnipro River for both 6-month and 9-month mean transit times are greatest in the winter months, when there is typically ice cover on the river. The greatest groundwater contribution comes in December, at 60% and 51% for the 6-month and 9-month calculations, respectively.

Generally, in most districts (except for Sviatoshynskyi), winter and fall are the seasons with the highest ratio of groundwater in the water supply. These are also the times when the groundwater contribution percentage to the Dnipro is the highest. As climate change progresses and winter ice cover continues to decrease [42], the timing of maximum and minimum precipitation amounts may shift, and the total yearly precipitation amount may shift [40]. Consequently, groundwater withdrawals for the municipal water supply could affect the flow of the Dnipro. Furthermore, if the discharge of the Dnipro River decreases as predicted by Didovets et al. [55], the river may depend more upon the groundwater contribution. While the estimated percent contribution of groundwater to the Dnipro River during this time agrees with previous findings, continued unpredictable precipitation may begin to impact this percentage.

## Conclusions

Tap-water surveys have been increasingly used to identify spatial and temporal patterns in municipal water sources. In Kyiv, where most of the city’s water comes from two rivers that are vulnerable to influences from climate change and pollution [59, 60], understanding the reliance on these sources in the context of climate change is critical. As the air and Dnipro River temperatures are increasing and the timing and amount of precipitation are already shifting, early preparation for disruption in water resources is necessary for effective water management.

The stable isotope values for tap water collected from November 2019 through December 2020 show a general seasonal trend for most of the districts, with more negative isotope values in the winter and less negative values in the summer. These patterns follow the general trends of the isotopic values from precipitation, though the actual numbers show a clear addition of groundwater. The groundwater percentage contribution also follows this general trend, with a higher percentage of groundwater used in the winter and a lower percentage in the summer for most of the districts. There is also a clear spatial trend, with a higher percentage of surface water used in the districts on the right bank and a lower percentage used in the districts on the left bank. Furthermore, there is a north-to-south trend on each bank, with the northern districts on the right bank using a higher percentage of surface water, while the northern districts on the left bank use a lower percentage of surface water. As climate change progresses, this can affect both the quantity and quality of surface water and groundwater, making it critical to assess sources of municipal water continually. Stable isotope ratios in tap water and the source waters provide a way to assess changes to the sources, reduce the uncertainty associated with physical measurements, and adapt the water supply accordingly. Samples from a finer geographical scale and across a longer temporal range will be necessary to confirm and expand on this dataset and to examine spatial and temporal variation within each district.

## Supplementary Information

Below is the link to the electronic supplementary material.

**Additional file 1: Table S1.** Tap water samples for the ten districts of Kyiv city, Boryspil, Boyarka, and Brovary. To differentiate unique locations while maintaining volunteer privacy, a number is given to the name when more than one sample location is used in one district. * = additional sample taken from outside of sampling period, not included on the map for spatial/temporal consideration but considered in calculated averages for the district. **Table S2.** Surface water samples from the Dnipro and Desenka Rivers at Kyiv and the Dnipro River at Cherkasy. **Table S3.** Groundwater samples from the ten districts in Kyiv.

## Data Availability

The data that support the findings of this study are available in the supplementary material of this article.
