# Supplementary material for: Variation of tap-water isotope ratios and municipal water sources across Kyiv city, Ukraine
Source: Discov Water. 2022 Nov 7;2(1):13. doi: 10.1007/s43832-022-00021-x (PMC9640821; doi:10.1007/s43832-022-00021-x)
Supplement: Supplementary file 1 — Additional file 1: Table S1. Tap water samples for the ten districts of Kyiv city, Boryspil, Boyarka, and Brovary. To differentiate unique locations while maintaining volunteer privacy, a number is given to the name when more than one sample location is used in one district. * = additional sample taken from outside of sampling period, not included on the map for spatial/temporal consideration but considered in calculated averages for the district. Table S2. Surface water samples from the Dnipro and Desenka Rivers at Kyiv and the Dnipro River at Cherkasy. Table S3. Groundwater samples from the ten districts in Kyiv. [file 43832_2022_21_MOESM1_ESM.docx]

| **Raion/City** | **Month** | ***δ*^2^H (‰ )** | **SD** | ***δ*^18^O (‰)** | **SD** |
| --- | --- | --- | --- | --- | --- |
| Dniprovskyi 1 | November 2019 | -76.4 | 0.20 | -10.3 | 0.07 |
| Dniprovskyi 2 | November 2019 | -73.8 | 0.11 | -11.0 | 0.03 |
| Darnytskyi 1 | November 2019 | -73.5 | 0.09 | -10.0 | 0.03 |
| Obolonskyi 1 | November 2019 | -62.2 | 0.09 | -8.2 | 0.07 |
| Podilskyi 1 | November 2019 | -67.6 | 0.15 | -8.8 | 0.05 |
| Shevchenkivskyi 1 | November 2019 | -71.5 | 0.08 | -9.4 | 0.01 |
| Solomianskyi 1 | November 2019 | -73.1 | 0.07 | -9.9 | 0.03 |
| Pecherskyi 1 | November 2019 | -73.6 | 0.23 | -10.7 | 0.12 |
| Holosiivskyi | November 2019 | -73.4 | 0.15 | -9.6 | 0.03 |
| Desnianskyi 1 | December 2019 | -79.0 | 0.12 | -10.6 | 0.13 |
| Dniprovskyi 3 | December 2019 | -74.7 | 0.06 | -10.1 | 0.05 |
| Dniprovskyi 1 | December 2019 | -76.3 | 0.11 | -10.3 | 0.11 |
| Dniprovskyi 4 | December 2019 | -75.8 | 0.27 | -10.3 | 0.02 |
| Podilskyi 2 | December 2019 | -76.0 | 0.06 | -10.2 | 0.02 |
| Podilskyi 3 | December 2019 | -76.5 | 0.15 | -10.5 | 0.15 |
| Podilskyi 1 | December 2019 | -67.3 | 0.16 | -8.7 | 0.09 |
| Sviatoshynskyi | December 2019 | -64.5 | 0.08 | -8.6 | 0.04 |
| Shevchenkivskyi 1 | December 2019 | -68.8 | 0.22 | -9.1 | 0.03 |
| Shevchenkivskyi 2 | December 2019 | -69.4 | 0.20 | -9.3 | 0.09 |
| Holosiivskyi | December 2019 | -76.2 | 0.19 | -10.5 | 0.08 |
| Boryspil | December 2019 | -83.6 | 0.14 | -11.5 | 0.05 |
| Boyarka | December 2019 | -76.9 | 0.11 | -11.2 | 0.08 |
| Brovary | December 2019 | -76.0 | 0.23 | -10.4 | 0.16 |
| Desnianskyi 1 | January 2020 | -78.6 | 0.08 | -10.3 | 0.04 |
| Dniprovskyi 1 | January 2020 | -76.4 | 0.09 | -10.4 | 0.05 |
| Dniprovskyi 4 | January 2020 | -76.6 | 0.07 | -10.4 | 0.06 |
| Obolonskyi 1 | January 2020 | -79.1 | 0.15 | -10.6 | 0.05 |
| Obolonskyi 2 | January 2020 | -70.1 | 0.07 | -9.6 | 0.04 |
| Podilskyi 1 | January 2020 | -69.9 | 0.23 | -9.7 | 0.08 |
| Sviatoshynskyi | January 2020 | -69.0 | 0.18 | -9.4 | 0.06 |
| Shevchenkivskyi 1 | January 2020 | -75.2 | 0.21 | -9.9 | 0.07 |
| Shevchenkivskyi 2 | January 2020 | -73.2 | 0.22 | -10.5 | 0.12 |
| Solomianskyi 1 | January 2020 | -77.6 | 0.11 | -11.0 | 0.06 |
| Pecherskyi 2 | January 2020 | -76.5 | 0.14 | -10.5 | 0.02 |
| Pecherskyi 3 | January 2020 | -76.6 | 0.15 | -10.6 | 0.06 |
| Holosiivskyi | January 2020 | -76.6 | 0.08 | -10.5 | 0.04 |
| Boryspil | January 2020 | -84.6 | 0.15 | -12.1 | 0.08 |
| Boyarka | January 2020 | -76.8 | 0.09 | -10.9 | 0.03 |
| Brovary | January 2020 | -76.0 | 0.08 | -10.4 | 0.12 |
| Dniprovskyi 4 | February 2020 | -77.2 | 0.20 | -10.6 | 0.07 |
| Dniprovskyi 5 | February 2020 | -77.3 | 0.24 | -10.4 | 0.06 |
| Dniprovskyi 6 | February 2020 | -77.0 | 0.22 | -11.1 | 0.07 |
| Dniprovskyi 7 | February 2020 | -77.7 | 0.09 | -10.5 | 0.04 |
| Darnytskyi 1 | February 2020 | -78.1 | 0.22 | -10.3 | 0.07 |
| Podilskyi 1 | February 2020 | -73.9 | 0.04 | -10.1 | 0.06 |
| Shevchenkivskyi 2 | February 2020 | -71.9 | 0.11 | -9.9 | 0.08 |
| Solomianskyi 1 | February 2020 | -77.4 | 0.23 | -10.7 | 0.05 |
| Holosiivskyi | February 2020 | -77.9 | 0.16 | -10.8 | 0.04 |
| Boryspil | February 2020 | -84.1 | 0.07 | -11.6 | 0.04 |
| Boyarka | February 2020 | -76.4 | 0.22 | -10.8 | 0.05 |
| Desnianskyi 2 | March 2020 | -78.5 | 0.20 | -10.3 | 0.05 |
| Dniprovskyi 4 | March 2020 | -76.4 | 0.35 | -10.6 | 0.12 |
| Dniprovskyi 7 | March 2020 | -76.2 | 0.17 | -10.5 | 0.16 |
| Obolonskyi 2 | March 2020 | -72.4 | 0.08 | -9.9 | 0.01 |
| Dniprovskyi 5 | March 2020 | -76.1 | 0.13 | -10.4 | 0.02 |
| Shevchenkivskyi 2 | March 2020 | -73.7 | 0.07 | -10.3 | 0.05 |
| Shevchenkivskyi 1 | March 2020 | -75.6 | 0.07 | -10.6 | 0.03 |
| Solomianskyi 2 | March 2020 | -77.0 | 0.08 | -10.7 | 0.01 |
| Pecherskyi 3 | March 2020 | -75.9 | 0.13 | -10.3 | 0.03 |
| Boryspil | March 2020 | -86.1 | 0.14 | -12.1 | 0.07 |
| Boyarka | March 2020 | -76.9 | 0.20 | -11.3 | 0.03 |
| Brovary | March 2020 | -77.7 | 0.21 | -10.6 | 0.03 |
| Desnianskyi 3 | April 2020 | -75.0 | 0.14 | -10.1 | 0.04 |
| Dniprovskyi 4 | April 2020 | -74.2 | 0.10 | -9.8 | 0.03 |
| Dniprovskyi 7 | April 2020 | -74.8 | 0.09 | -10.2 | 0.08 |
| Dniprovskyi 5 | April 2020 | -74.1 | 0.15 | -10.0 | 0.08 |
| Obolonskyi 2 | April 2020 | -70.8 | 0.10 | -10.0 | 0.06 |
| Podilskyi 1 | April 2020 | -71.3 | 0.45 | -9.9 | 0.06 |
| Sviatoshynskyi | April 2020 | -70.5 | 0.23 | -9.6 | 0.07 |
| Shevchenkivskyi 2 | April 2020 | -71.2 | 0.15 | -9.8 | 0.05 |
| Shevchenkivskyi 1 | April 2020 | -72.5 | 0.12 | -9.7 | 0.04 |
| Solomianskyi 2 | April 2020 | -74.4 | 0.10 | -10.2 | 0.08 |
| Pecherskyi 3 | April 2020 | -74.3 | 0.13 | -9.9 | 0.03 |
| Boryspil | April 2020 | -83.7 | 0.22 | -11.5 | 0.08 |
| Boyarka | April 2020 | -76.7 | 0.22 | -10.9 | 0.03 |
| Brovary | April 2020 | -74.4 | 0.16 | -10.1 | 0.02 |
| Desnianskyi 2 | May 2020 | -78.1 | 0.10 | -10.2 | 0.04 |
| Desnianskyi 3 | May 2020 | -72.3 | 0.13 | -10.0 | 0.05 |
| Dniprovskyi 7* | May 2020 | -67.0 | 0.13 | -9.1 | 0.09 |
| Dniprovskyi 4 | May 2020 | -71.6 | 0.06 | -9.6 | 0.06 |
| Dniprovskyi 7 | May 2020 | -71.5 | 0.16 | -9.7 | 0.19 |
| Dniprovskyi 5 | May 2020 | -71.8 | 0.19 | -9.6 | 0.12 |
| Obolonskyi 2 | May 2020 | -68.2 | 0.07 | -9.1 | 0.05 |
| Podilskyi 1 | May 2020 | -66.7 | 0.14 | -8.8 | 0.03 |
| Shevchenkivskyi 1 | May 2020 | -70.7 | 0.12 | -9.5 | 0.04 |
| Shevchenkivskyi 2 | May 2020 | -68.2 | 0.14 | -9.3 | 0.04 |
| Shevchenkivskyi 1* | May 2020 | -71.8 | 0.26 | -9.9 | 0.04 |
| Solomianskyi 2 | May 2020 | -72.2 | 0.14 | -9.9 | 0.07 |
| Boryspil | May 2020 | -84.3 | 0.22 | -11.7 | 0.07 |
| Boyarka | May 2020 | -76.7 | 0.25 | -10.8 | 0.05 |
| Brovary | May 2020 | -72.0 | 0.10 | -9.8 | 0.04 |
| Desnianskyi 3 | June 2020 | -69.0 | 0.10 | -9.4 | 0.06 |
| Desnianskyi 2* | June 2020 | -76.5 | 0.17 | -10.0 | 0.11 |
| Dniprovskyi 4 | June 2020 | -66.8 | 0.03 | -9.3 | 0.04 |
| Dniprovskyi 5 | June 2020 | -66.5 | 0.23 | -9.0 | 0.04 |
| Obolonskyi 2 | June 2020 | -66.2 | 0.22 | -8.7 | 0.03 |
| Podilskyi 1 | June 2020 | -67.2 | 0.03 | -9.2 | 0.03 |
| Sviatoshynskyi | June 2020 | -66.9 | 0.10 | -9.0 | 0.06 |
| Shevchenkivskyi 2 | June 2020 | -66.6 | 0.34 | -8.9 | 0.06 |
| Shevchenkivskyi 1 | June 2020 | -65.7 | 0.28 | -8.9 | 0.06 |
| Solomianskyi 2 | June 2020 | -67.2 | 0.09 | -9.4 | 0.06 |
| Boryspil | June 2020 | -85.7 | 0.05 | -11.8 | 0.05 |
| Brovary | June 2020 | -67.3 | 0.11 | -9.0 | 0.02 |
| Desnianskyi 2 | July 2020 | -77.5 | 0.23 | -10.1 | 0.08 |
| Dniprovskyi 7 | July 2020 | -63.6 | 0.16 | -8.1 | 0.12 |
| Dniprovskyi 4 | July 2020 | -61.0 | 0.19 | -7.9 | 0.07 |
| Dniprovskyi 5 | July 2020 | -64.0 | 0.18 | -8.2 | 0.04 |
| Darnytskyi 1 | July 2020 | -61.3 | 0.18 | -8.0 | 0.05 |
| Obolonskyi 2 | July 2020 | -61.6 | 0.23 | -8.1 | 0.08 |
| Podilskyi 1 | July 2020 | -61.5 | 0.21 | -8.2 | 0.16 |
| Sviatoshynskyi | July 2020 | -58.1 | 0.24 | -7.5 | 0.05 |
| Shevchenkivskyi 2 | July 2020 | -61.4 | 0.30 | -8.1 | 0.07 |
| Solomianskyi 2 | July 2020 | -64.4 | 0.19 | -8.0 | 0.03 |
| Pecherskyi 4 | July 2020 | -64.7 | 0.22 | -8.4 | 0.05 |
| Boryspil | July 2020 | -84.5 | 0.21 | -11.7 | 0.04 |
| Boyarka | July 2020 | -76.8 | 0.13 | -10.9 | 0.03 |
| Boyarka* | July 2020 | -76.8 | 0.32 | -10.9 | 0.04 |
| Brovary | July 2020 | -69.3 | 0.17 | -9.4 | 0.02 |
| Desnianskyi 2 | August 2020 | -74.0 | 0.17 | -9.6 | 0.02 |
| Dniprovskyi 7 | August 2020 | -61.7 | 0.23 | -8.0 | 0.08 |
| Dniprovskyi 4 | August 2020 | -68.3 | 0.05 | -9.0 | 0.04 |
| Dniprovskyi 5 | August 2020 | -63.0 | 0.09 | -8.2 | 0.03 |
| Darnytskyi 1 | August 2020 | -62.5 | 0.06 | -8.2 | 0.06 |
| Obolonskyi 2 | August 2020 | -57.4 | 0.17 | -7.8 | 0.03 |
| Sviatoshynskyi | August 2020 | -57.6 | 0.20 | -7.5 | 0.06 |
| Shevchenkivskyi 2 | August 2020 | -61.1 | 0.37 | -7.8 | 0.05 |
| Solomianskyi 2 | August 2020 | -62.2 | 0.09 | -8.0 | 0.02 |
| Boryspil | August 2020 | -84.8 | 0.16 | -11.8 | 0.04 |
| Boyarka | August 2020 | -77.3 | 0.23 | -10.9 | 0.11 |
| Brovary | August 2020 | -62.3 | 0.09 | -8.2 | 0.02 |
| Desnianskyi 2 | September 2020 | -77.2 | 0.21 | -10.0 | 0.06 |
| Dniprovskyi 4 | September 2020 | -68.2 | 0.05 | -9.0 | 0.05 |
| Darnytskyi 1 | September 2020 | -67.5 | 0.20 | -8.9 | 0.04 |
| Obolonskyi 2 | September 2020 | -59.4 | 0.09 | -7.9 | 0.02 |
| Podilskyi 1 | September 2020 | -55.8 | 0.14 | -7.3 | 0.03 |
| Sviatoshynskyi | September 2020 | -58.2 | 0.14 | -7.6 | 0.03 |
| Shevchenkivskyi 2 | September 2020 | -64.9 | 0.15 | -8.6 | 0.01 |
| Solomianskyi 2 | September 2020 | -67.4 | 0.14 | -8.3 | 0.05 |
| Boryspil | September 2020 | -85.5 | 0.24 | -11.7 | 0.09 |
| Brovary | September 2020 | -61.5 | 0.05 | -8.0 | 0.02 |
| Desnianskyi 2 | October 2020 | -77.2 | 0.07 | -9.9 | 0.04 |
| Dniprovskyi 4 | October 2020 | -70.3 | 0.32 | -10.2 | 0.06 |
| Dniprovskyi 5 | October 2020 | -69.5 | 0.09 | -9.3 | 0.05 |
| Darnytskyi 1 | October 2020 | -68.9 | 0.09 | -9.0 | 0.03 |
| Obolonskyi 2 | October 2020 | -61.1 | 0.18 | -8.1 | 0.02 |
| Podilskyi 1 | October 2020 | -70.4 | 0.08 | -9.6 | 0.05 |
| Sviatoshynskyi | October 2020 | -59.6 | 0.18 | -7.9 | 0.04 |
| Shevchenkivskyi 2 | October 2020 | -67.2 | 0.24 | -9.0 | 0.14 |
| Solomianskyi 2 | October 2020 | -69.3 | 0.11 | -9.2 | 0.06 |
| Boryspil | October 2020 | -84.5 | 0.12 | -11.8 | 0.06 |
| Boyarka* | October 2020 | -76.9 | 0.12 | -10.9 | 0.03 |
| Boyarka | October 2020 | -77.1 | 0.16 | -11.1 | 0.04 |
| Brovary | October 2020 | -68.0 | 0.08 | -9.0 | 0.04 |
| Desnianskyi 2 | November 2020 | -77.6 | 0.11 | -10.1 | 0.03 |
| Dniprovskyi 4 | November 2020 | -70.6 | 0.09 | -9.5 | 0.05 |
| Dniprovskyi 5 | November 2020 | -70.8 | 0.11 | -9.6 | 0.03 |
| Darnytskyi 2 | November 2020 | -87.2 | 0.25 | -12.0 | 0.11 |
| Darnytskyi 1 | November 2020 | -70.8 | 0.08 | -9.6 | 0.03 |
| Obolonskyi 2 | November 2020 | -59.8 | 0.09 | -8.1 | 0.03 |
| Podilskyi 1 | November 2020 | -72.8 | 0.05 | -9.8 | 0.02 |
| Sviatoshynskyi | November 2020 | -62.4 | 0.19 | -8.5 | 0.04 |
| Shevchenkivskyi 2 | November 2020 | -69.1 | 0.21 | -9.3 | 0.04 |
| Solomianskyi 2 | November 2020 | -70.5 | 0.11 | -9.5 | 0.07 |
| Boryspil | November 2020 | -84.6 | 0.10 | -11.6 | 0.04 |
| Boyarka | November 2020 | -76.7 | 0.17 | -10.8 | 0.04 |
| Brovary | November 2020 | -71.0 | 0.13 | -9.6 | 0.05 |
| Desnianskyi 2 | December 2020 | -78.2 | 0.15 | -10.1 | 0.05 |
| Dniprovskyi 4 | December 2020 | -74.1 | 0.18 | -10.2 | 0.06 |
| Dniprovskyi 5 | December 2020 | -74.2 | 0.26 | -10.2 | 0.03 |
| Dniprovskyi 7 | December 2020 | -73.8 | 0.06 | -9.9 | 0.04 |
| Darnytskyi 2 | December 2020 | -87.9 | 0.11 | -11.9 | 0.03 |
| Darnytskyi 1 | December 2020 | -74.0 | 0.10 | -10.1 | 0.05 |
| Obolonskyi 2 | December 2020 | -63.8 | 0.11 | -8.9 | 0.02 |
| Podilskyi 1 | December 2020 | -63.8 | 0.13 | -8.8 | 0.05 |
| Sviatoshynskyi | December 2020 | -63.9 | 0.08 | -8.8 | 0.04 |
| Shevchenkivskyi 2 | December 2020 | -72.1 | 0.09 | -9.7 | 0.04 |
| Solomianskyi 2 | December 2020 | -74.5 | 0.05 | -10.2 | 0.08 |
| Boryspil | December 2020 | -84.1 | 0.14 | -11.7 | 0.07 |
| Boyarka | December 2020 | -77.0 | 0.10 | -11.0 | 0.04 |
| Brovary | December 2020 | -75.5 | 0.24 | -10.5 | 0.04 |

Supplementary Table 1. Tap water samples for the ten districts of Kyiv city, Boryspil, Boyarka, and Brovary. To differentiate unique locations while maintaining volunteer privacy, a number is given to the name when more than one sample location is used in one district. * = additional sample taken from outside of sampling period, not included on the map for spatial/temporal consideration but considered in calculated averages for the district.

| **At Kyiv** | | | | | |
| --- | --- | --- | --- | --- | --- |
| **Location** | **Month** | ***δ*^2^H (‰)** | **SD** | ***δ*^18^O (‰)** | **SD** |
| Dnipro River | December 2019 | -66.7 | 0.12 | -9.0 | 0.03 |
| Desenka River | December 2019 | -61.6 | 0.21 | -7.8 | 0.02 |
| Dnipro River | January 2020 | -70.2 | 0.16 | -9.6 | 0.08 |
| Desenka River | January 2020 | -63.2 | 0.11 | -8.2 | 0.10 |
| Dnipro River | March 2020 | -73.2 | 0.22 | -10.2 | 0.01 |
| Dnipro River | May 2020 | -69.1 | 0.12 | -9.3 | 0.04 |
| Dnipro River | June 2020 | -66.0 | 0.16 | -9.0 | 0.08 |
| Dnipro River | July 2020 | -61.9 | 0.13 | -8.2 | 0.03 |
| Desenka River | July 2020 | -61.6 | 0.14 | -7.9 | 0.06 |
| Dnipro River | August 2020 | -59.0 | 0.15 | -7.5 | 0.05 |
| Desenka River | August 2020 | -59.4 | 0.23 | -7.7 | 0.01 |
| Dnipro River | September 2020 | -57.6 | 0.08 | -7.4 | 0.06 |
| Desenka River | September 2020 | -58.7 | 0.16 | -7.5 | 0.08 |
| Dnipro River | October 2020 | -60.0 | 0.08 | -7.9 | 0.04 |
| Desenka River | October 2020 | -58.2 | 0.08 | -7.5 | 0.05 |
| Dnipro River | November 2020 | -62.1 | 0.12 | -8.5 | 0.04 |
| Desenka River | November 2020 | -58.9 | 0.20 | -7.6 | 0.04 |
| Dnipro River | December 2020 | -69.8 | 0.32 | -9.7 | 0.04 |
| Desenka River | December 2020 | -60.4 | 0.25 | -8.1 | 0.07 |
| **At Cherkasy** | | | | | |
| **Location** | **Month** | ***δ*^2^H (‰)** | **SD** | ***δ*^18^O (‰)** | **SD** |
| Dnipro River | March 2020 | -70.4 | 0.21 | -9.8 | 0.05 |
| Dnipro River | April 2020 | -70.5 | 0.14 | -9.8 | 0.04 |
| Dnipro River | May 2020 | -69.5 | 0.18 | -9.5 | 0.01 |
| Dnipro River | June 2020 | -61.0 | 0.17 | -7.6 | 0.06 |
| Dnipro River | August 2020 | -56.3 | 0.38 | -7.3 | 0.02 |
| Dnipro River | October 2020 | -56.8 | 0.27 | -7.2 | 0.04 |
| Dnipro River | November 2020 | -57.0 | 0.41 | -7.3 | 0.06 |
| Dnipro River | December 2020 | -60.7 | 0.21 | -7.9 | 0.08 |

Supplementary Table 2. Surface water samples from the Dnipro and Desenka Rivers at Kyiv and the Dnipro River at Cherkasy.

| **Raion/City** | **Month** | ***δ*^2^H (‰)** | **SD** | ***δ*^18^O (‰)** | **SD** |
| --- | --- | --- | --- | --- | --- |
| Dniprovskyi | January 2020 | -77.8 | 0.15 | -11.1 | 0.04 |
| Darnytskyi | January 2020 | -89.5 | 0.16 | -12.2 | 0.04 |
| Obolonskyi | January 2020 | -77.2 | 0.01 | -10.8 | 0.12 |
| Sviatoshynskyi | January 2020 | -80.9 | 0.13 | -11.7 | 0.09 |
| Podilskyi | February 2020 | -80.4 | 0.05 | -10.6 | 0.05 |
| Desnianskyi | March 2020 | -79.8 | 0.20 | -10.6 | 0.05 |
| Dniprovskyi | March 2020 | -77.8 | 0.21 | -11.2 | 0.05 |
| Shevchenkivskyi | March 2020 | -86.2 | 0.11 | -12.0 | 0.03 |
| Shevchenkivskyi | April 2020 | -86.1 | 0.17 | -12.1 | 0.05 |
| Desnianskyi | May 2020 | -80.0 | 0.09 | -10.2 | 0.08 |
| Dniprovskyi | May 2020 | -77.6 | 0.18 | -10.9 | 0.05 |
| Shevchenkivskyi | May 2020 | -86.3 | 0.16 | -11.9 | 0.08 |
| Desnianskyi | June 2020 | -80.0 | 0.23 | -10.3 | 0.06 |
| Dniprovskyi | June 2020 | -78.0 | 0.25 | -10.8 | 0.03 |
| Shevchenkivskyi | June 2020 | -86.3 | 0.12 | -12.0 | 0.08 |
| Desnianskyi | July 2020 | -79.7 | 0.19 | -10.3 | 0.05 |
| Dniprovskyi | July 2020 | -77.8 | 0.16 | -10.8 | 0.03 |
| Obolonskyi | July 2020 | -79.7 | 0.20 | -10.7 | 0.13 |
| Podilskyi | July 2020 | -81.6 | 0.17 | -10.5 | 0.08 |
| Sviatoshynskyi | July 2020 | -80.2 | 0.20 | -11.4 | 0.08 |
| Shevchenkivskyi | July 2020 | -85.9 | 0.17 | -12.0 | 0.07 |
| Solomianskyi 1 | July 2020 | -84.2 | 0.17 | -11.7 | 0.07 |
| Pecherskyi 1 | July 2020 | -78.4 | 0.10 | -10.8 | 0.10 |
| Desnianskyi | August 2020 | -79.8 | 0.08 | -10.7 | 0.04 |
| Dniprovskyi | August 2020 | -78.0 | 0.18 | -10.8 | 0.04 |
| Darnytskyi | August 2020 | -88.1 | 0.06 | -11.8 | 0.02 |
| Obolonskyi | August 2020 | -81.0 | 0.31 | -10.8 | 0.06 |
| Podilskyi | August 2020 | -80.7 | 0.21 | -10.6 | 0.06 |
| Sviatoshynskyi | August 2020 | -80.2 | 0.21 | -11.6 | 0.08 |
| Shevchenkivskyi | August 2020 | -85.4 | 0.28 | -12.0 | 0.04 |
| Solomianskyi 1 | August 2020 | -81.3 | 0.33 | -11.5 | 0.05 |
| Solomianskyi 2 | August 2020 | -83.1 | 0.27 | -11.1 | 0.02 |
| Pecherskyi 1 | August 2020 | -78.4 | 0.40 | -11.0 | 0.05 |
| Desnianskyi | September 2020 | -80.0 | 0.22 | -10.4 | 0.06 |
| Dniprovskyi | September 2020 | -77.9 | 0.12 | -10.8 | 0.04 |
| Darnytskyi | September 2020 | -87.9 | 0.15 | -11.9 | 0.08 |
| Obolonskyi | September 2020 | -81.2 | 0.10 | -10.9 | 0.04 |
| Podilskyi | September 2020 | -81.0 | 0.07 | -11.0 | 0.07 |
| Sviatoshynskyi | September 2020 | -80.3 | 0.14 | -11.3 | 0.07 |
| Shevchenkivskyi 2 | September 2020 | -74.3 | 0.32 | -10.2 | 0.06 |
| Shevchenkivskyi 1 | September 2020 | -86.6 | 0.09 | -11.5 | 0.08 |
| Solomianskyi 1 | September 2020 | -80.7 | 0.11 | -11.2 | 0.05 |
| Solomianskyi 2 | September 2020 | -82.8 | 0.14 | -11.1 | 0.01 |
| Pecherskyi 1 | September 2020 | -78.6 | 0.11 | -11.2 | 0.04 |
| Holosiivskyi | September 2020 | -83.1 | 0.13 | -11.0 | 0.03 |
| Desnianskyi | October 2020 | -80.1 | 0.11 | -10.5 | 0.07 |
| Darnytskyi | October 2020 | -88.0 | 0.05 | -11.8 | 0.09 |
| Obolonskyi | October 2020 | -81.2 | 0.10 | -10.9 | 0.05 |
| Podilskyi | October 2020 | -81.6 | 0.35 | -11.2 | 0.14 |
| Sviatoshynskyi | October 2020 | -79.8 | 0.10 | -11.3 | 0.04 |
| Shevchenkivskyi 2 | October 2020 | -71.2 | 0.16 | -9.6 | 0.02 |
| Shevchenkivskyi 1 | October 2020 | -86.2 | 0.21 | -12.1 | 0.05 |
| Solomianskyi 1 | October 2020 | -81.0 | 0.14 | -11.4 | 0.02 |
| Solomianskyi 2 | October 2020 | -82.0 | 0.20 | -11.0 | 0.05 |
| Pecherskyi 2 | October 2020 | -74.5 | 0.31 | -10.2 | 0.05 |
| Pecherskyi 1 | October 2020 | -79.1 | 0.19 | -10.7 | 0.07 |
| Desnianskyi | November 2020 | -79.9 | 0.19 | -10.5 | 0.07 |
| Dniprovskyi | November 2020 | -77.6 | 0.24 | -10.8 | 0.05 |
| Obolonskyi | November 2020 | -81.6 | 0.03 | -10.7 | 0.06 |
| Podilskyi | November 2020 | -81.1 | 0.23 | -10.9 | 0.04 |
| Sviatoshynskyi | November 2020 | -80.3 | 0.14 | -11.4 | 0.07 |
| Shevchenkivskyi 2 | November 2020 | -74.7 | 0.17 | -10.2 | 0.05 |
| Shevchenkivskyi 1 | November 2020 | -86.5 | 0.24 | -12.1 | 0.11 |
| Solomianskyi 1 | November 2020 | -81.6 | 0.10 | -11.6 | 0.06 |
| Pecherskyi 2 | November 2020 | -73.9 | 0.05 | -10.2 | 0.03 |
| Pecherskyi 1 | November 2020 | -78.8 | 0.20 | -11.1 | 0.06 |
| Holosiivskyi | November 2020 | -82.4 | 0.20 | -10.5 | 0.04 |
| Desnianskyi | December 2020 | -79.9 | 0.16 | -10.4 | 0.03 |
| Dniprovskyi | December 2020 | -77.9 | 0.14 | -10.8 | 0.06 |
| Obolonskyi | December 2020 | -81.0 | 0.24 | -10.6 | 0.03 |
| Podilskyi | December 2020 | -80.7 | 0.22 | -10.8 | 0.03 |
| Sviatoshynskyi | December 2020 | -80.0 | 0.12 | -11.3 | 0.07 |
| Shevchenkivskyi 2 | December 2020 | -74.6 | 0.17 | -10.2 | 0.08 |
| Shevchenkivskyi 1 | December 2020 | -85.9 | 0.04 | -11.8 | 0.02 |
| Solomianskyi 1 | December 2020 | -80.8 | 0.22 | -11.3 | 0.03 |
| Pecherskyi 2 | December 2020 | -73.9 | 0.11 | -10.1 | 0.05 |
| Pecherskyi 1 | December 2020 | -78.4 | 0.17 | -11.0 | 0.03 |
| Holosiivskyi | December 2020 | -82.9 | 0.07 | -10.8 | 0.05 |

Supplementary Table 3. Groundwater samples from the ten districts in Kyiv.
